# Prevalence and Reinfection Rates of* Schistosoma mansoni* and Praziquantel Efficacy against the Parasite among Primary School Children in Sanja Town, Northwest Ethiopia

**DOI:** 10.1155/2019/3697216

**Published:** 2019-04-24

**Authors:** Eden Woldegerima, Abebe Genetu Bayih, Yalewayker Tegegne, Mulugeta Aemero, Ayalew Jejaw Zeleke

**Affiliations:** ^1^University of Gondar Referral Hospital, College of Medicine and Health Sciences, University of Gondar, Gondar, Ethiopia; ^2^Department of Medical Parasitology, School of Biomedical and Laboratory Sciences, College of Medicine and Health Sciences, University of Gondar, Gondar, Ethiopia; ^3^Armauer Hansen Research Institute, Addis Ababa, Ethiopia

## Abstract

**Background:**

Schistosomiasis is among the most widespread chronic infections in the world. The magnitude of the infection may show variations across different areas with respect to time. Praziquantel is a first line drug of choice for the treatment of schistosomiasis although its low cure rate has been reported in different parts of the world. Thus, an assessment of the magnitude of the diseases, the efficacy of currently available drugs, and reinfection rates is crucial.

**Objective:**

Our principal objective is to determine the prevalence and reinfection rates of* Schistosoma mansoni *and to evaluate the efficacy of PZQ against* Schistosoma mansoni*.

**Method:**

A school-based cross-sectional study was conducted on Sanja Elementary Schools, Sanja town, northwest Ethiopia. Stool specimens were examined using Kato-Katz method. Schoolchildren who tested positive for intestinal schistosomiasis and fulfilled the inclusion criteria took part in the efficacy and reinfection study. Positive participants were treated with 40 mg/kg of Praziquantel. Cure and egg reduction rates were evaluated three weeks after treatment. The intensity of infection was determined following the WHO's guideline. Moreover, the reinfection rate of those who were cured was evaluated after a six-month posttreatment period. Data were analyzed using SPSS version 20.

**Results:**

At baseline, 130 (35%) of the 372 schoolchildren were found infected with* Schistosoma mansoni. *Out of the 130 infected schoolchildren, 112 (86.2%) had moderate infection intensity. Among the* S. mansoni* positive schoolchildren, 80 were included as study participants for the evaluation of PZQ efficacy, based on the inclusion criteria established by WHO. The cure and egg reduction rates were found to be 90% (72/80) and 99.5%, respectively. Of the seventy-two schoolchildren considered for the determination of reinfection rate, after 6 months of posttreatment, 13.9% were found to be reinfected.

**Conclusion:**

The schoolchildren in the three primary schools of Sanja are at moderate risk of the infection caused by* S. mansoni.* Although the therapeutic potency of PZQ at 40 mg/kg was efficient against* S. mansoni*, a high rate of reinfection was reported in the study site, suggesting the need for integrated schistosomiasis control measures.

## 1. Background

Schistosomiasis is a complex of acute and chronic parasitic infections caused by blood flukes of the genus* Schistosoma.* The two forms of the disease are known as urogenital and intestinal schistosomiasis [1]. The life cycle of* Schistosoma* parasites is completed in two different hosts, namely, humans and freshwater snails. Aquatic* Biomphalaria*,* Bulinus*, and amphibious* Oncomelania* snails are the intermediate hosts of* Schistosoma mansoni*,* Schistosoma haematobium,* and* Schistosoma japonicum,* respectively [2].* Schistosoma* parasites multiply in snails, the intermediate hosts. Humans that come into contact with fresh water containing snails are at risk of infection [3, 4].

The recent WHO report on disease burden estimated that more than 200,000 deaths per year are due to schistosomiasis in sub-Saharan Africa. Moreover, Schistosomiasis is prevalent in tropical and subtropical areas, especially in poor communities without access to safe drinking water and adequate sanitation. In 2014, 91.4% of people estimated to require treatment for schistosomiasis lived in the African region [5].

The control of intestinal schistosomiasis is mainly based on Praziquantel (PZQ) treatment, health education, and improved sanitation. School or community-based mass drug administration (MDA), which uses Praziquantel, is the major control strategy for schistosomiasis. Praziquantel, which is effective and safe, is the recommended treatment against all forms of schistosomiasis. An extensive use of PZQ worldwide may lead to a growing concern on its effectiveness, while some regions of the world report its low cure rate [6–8]. There is therefore a need for a regular monitoring of the drug under global pressure on its use. Moreover, reinfection has been reported rapidly after mass drug administrations (MDA) [9].

Schistosomiasis is the most common parasitic infection in Ethiopia, one of the poorest sub-Saharan African countries, which is within the WHO list of neglected tropical diseases (NTDs) [10]. The prevalence of the disease reported to be from 24 to 89.9% in different parts of the country and it is high among schoolchildren [4, 11–14]. In 2013, extremely high prevalence (89.9%) of* S. mansoni* was reported among schoolchildren in Sanja town, where the current study is carried out [4]. This report probably represented the highest figure that had ever published in history. Thus, it called for immediate implementation of different interventions like health education and Praziquantel mass drug administration. However, there is scarcity of evidences about the effectiveness of the intervention using chemotherapy in Sanja town in particular and in Ethiopia in general, even if the prevalence is high across different parts of the country.

In Ethiopia, the MDA strategy mainly focuses on school-aged children (SAC) via a yearly school-based treatment that started in 2015 and currently targets 6.4 million children in endemic areas nationwide. The government plans to achieve the elimination of schistosomiasis-related morbidity by 2020 [15]. Therefore, in line with the WHO (World Health Organization) recommendations, assessing treatment efficacy, reinfection rates, and the effect of MDA campaigns on infection prevalence is crucial. This may inform the policy makers of the country about the usefulness of MDA as a tool to achieve the elimination of schistosomiasis-related morbidity within the anticipated time [16]. Therefore, this study aimed to assess the prevalence and reinfection rates of* S. mansoni* and determine PZQ treatment efficacy against the parasite among primary school children in Sanja area, northwest Ethiopia.

## 2. Materials and Methods

### 2.1. Study Setting

**Figure 1:**
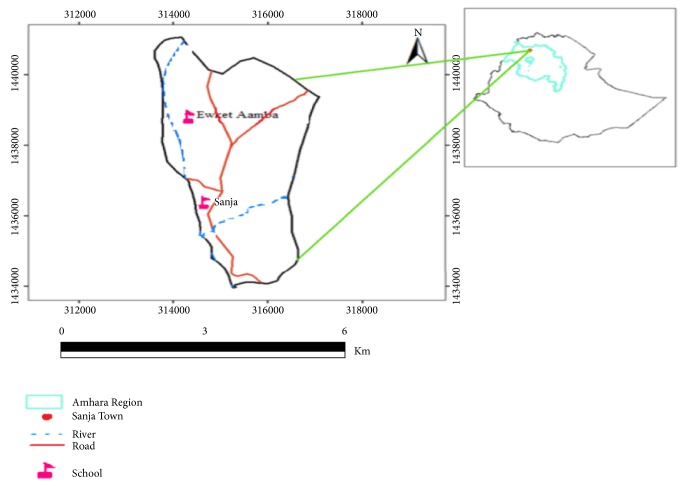
Maps of study area.

The study was conducted in Sanja area, located 65 km northwest of Gondar town and 792 km from Addis Ababa (Figure 1). With an estimated population of 26,000, Sanja is on an altitude of 1800 m above sea level, and its annual rainfall and average temperature range from 800 to 1800 mm and 25°C to 42°C, respectively [17]. Two health institutions (a health center and a hospital) are giving health care services for the population of the town and the surrounding areas. The study was conducted in schoolchildren aged 9-14 years. There are three elementary schools in the area, namely, Ewketamba, Sanja, and Hidassie. About 2811 students (1481 females and 1330 males) were attending their education during the study period in the area. A river and a stream, namely, Sanja and Maho, respectively, traverse the town. These water sources are used for washing, bathing, and other domestic and recreational purposes and may constitute the major sources of* S. mansoni* infection.

### 2.2. Study Design, Period, and Sample Size

A follow-up cross-sectional study was carried out on schoolchildren in Sanja, northwest Ethiopia, from December 2017 to May 2018. The required sample size was determined by using the single population proportion formula with the following assumptions: prevalence (p) of 50% to increase sample size, 95% confidence level, 5% margin of error, and a sample estimation correction formula were considered. Moreover, a 10% nonresponse rate was added and the final sample size (n) was =372. To select the study participants, the students were fist stratified according to their grade level (Grades 1 to Grade 8). The allocation was proportional to the number of students in each grade in all the three schools. Finally, the children were selected by the systematic sampling technique using class rosters as the sampling frame. Children who had taken antihelminthic drugs six months before the study, were in severe medical conditions, had liquid or diarrheal stool specimen, and vomited within 4 hours after drug administration were excluded.

### 2.3. Data Collection and Processing

#### 2.3.1. Questionnaire Survey

A pretested questionnaire written in Amharic (the local language) was employed to collect data using the interview technique. Data collectors were assigned to each school to conduct the survey and supervised by the principal investigator. Explanation about the aim of the research was given to the children and their teachers at each spot before the interview.

#### 2.3.2. Baseline Parasitological Survey

A single stool specimen of about 4g was collected from each study participant in the schools. A clean, dry, and leak proof container was used to collect a stool specimen, and the container for every child was labeled with a unique ID number and stool samples were transported to the laboratory of Sanja hospital. Each stool specimen was examined using two Kato-Katz preparations on a template holding 41.7 mg of stool. The slides were left for 24 hours for them to clear for easy visualization of* S. mansoni* eggs. After 24 hours, the smears were examined for eggs of* S. mansoni,* and the intensity of infections were classified as light (<100epg), moderate (100-400 epg), and heavy (>400 epg) per the threshold set by WHO [18]. Results of the laboratory investigation were recorded on reporting sheets prepared for reporting results. In addition, infection intensity of S*. mansoni* was calculated by multiplying the total number of eggs counted by 24, which gives eggs per gram (epg) of the stool.

### 2.4. Drug Administration

Each child was given a light snack (e.g., a slice of bread or a biscuit) before the drug was administered and took the same batch and brand of PZQ (Biltricide). The drug was within its expiry date and properly stored. The nurses gave the tablets under direct observation, and each child was kept under observation for approximately 4 hours. The children remained at school and continued their usual activities, but they were asked to rapidly report any side effect to a member of the investigation team [19].

### 2.5. Follow-Up Survey

Children who had a positive specimen and treated by PZQ at baseline were requested to provide a second specimen at 21 days. Schools were followed-up in the same order as in the baseline survey. Children who do not attend school on the follow-up day or do not bring a specimen were followed-up 1 or 2 days later. The laboratory method used in the baseline survey was used in the follow-up survey [19].

### 2.6. Reinfection Assessment

Children who tested positive during the baseline survey and negative following 14 to 21 days of posttreatment were tested 6 months after posttreatment to assess the reinfection rate.

### 2.7. Data Management and Analysis

Data was checked for its completeness before entering for analysis. Then it was cleaned, edited, and entered into SPSS version 20.0 for analysis. The overall PZQ efficacy was determined and recorded through the help of descriptive method of data analysis. Cure rate (CR) and egg reduction rate (ERR) were used to assess the drug efficacy as it is described before [19].* P*-value less than 0.05 was taken to determine as statistical significance associations at 95% CI.

(1) CR=Number of negative children after treatment who were positive at baselinenumber of positive children before treatment×100ERR=1−Arithmetic mean egg counts at follow upArithmetic mean egg counts at baseline×100

### 2.8. Ethical Considerations

Ethical approval was obtained from research and ethics committee of the School of Biomedical and Laboratory Sciences. Informed written consent was also sought from study participant's parent or guardian. An assent was also taken from the schoolchildren ≥12 years of old. All* S. mansoni* infected study subjects were treated with PZQ and other children who were positive for other intestinal parasitic infection were treated by linking them to Sanja hospital.

## 3. Results

### 3.1. Sociodemographic and Behavioral Characteristics of Study Participants

Three hundred seventy-two schoolchildren were recruited during the study. The mean age of study participants was 11.76 years (SD=±1.66). The proportion of male to female respondents was almost equal (1:1). As far as the characteristics of the study participants is concerned, 82.5%, 70.2%, and 49.2% of the study participants had habit of washing or bathing in rivers, open field defecation, and travel history to irrigation sites, respectively (Table 1).

### 3.2. Prevalence of* S. mansoni* Infection

The prevalence of* S. mansoni* was found to be 35% (130/372). Light, moderate, and heavy intensity of* S. mansoni *infections were detected in 0%, 86.2%, and 13.8% of the schoolchildren, respectively (Table 2).

### 3.3. Parasitological Cure and Egg Reduction Rates

All (130) infected school age children were treated with PZQ. However, twenty-three vomited within four hours, ten refused to participate during posttreatment, and seventeen were absent from school during the follow-up period. Thus, 80* S. mansoni positive *children were included in the drug efficacy study. Twenty-one days after treatment with PZQ at 40 mg/Kg body weight, stool samples were collected and examined using Kato-Katz. The overall cure rate of* S. mansoni* infected individuals was 90% (72/80) following the posttreatment. The cure rate was not significantly different among sexes and age groups* P*= >.05). The overall pretreatment arithmetic mean egg intensity (AMI) of infection was 680.31epg. However, it fell to 1.85 epg of stool three weeks after treatment, and the reduction was statistically significant (*P*-value=.000). Hence, the overall egg reduction rate was 99.55% (Table 3).

### 3.4. Reinfection Rate of* S. mansoni*

Seventy-two of the 80 school age children were cured on the 21st day following PZQ treatment. Therefore, reinfection rate was assessed in 72 schoolchildren six months after treatment. And 62 (86.1%) of them remained negative, while 13.9% were found reinfected within six months. All reinfection cases were in the light infection category. The reinfection rate showed a significant inverse association with age, that is, as age increased reinfection rate decreased. Moreover, although it was not statistically significant, higher reinfection rate was observed in males than females (Table 4).

## 4. Discussion

Schistosomiasis remains one of the most important but neglected tropical diseases in Ethiopia [10]. The rate of* S. mansoni* infection among schoolchildren in the study area was 35% (95% CI, 30.1-39.8%), a relatively moderate prevalence of* S. mansoni* infection. The finding is in agreement with those of studies carried out in Timuga (34%) [20] and Wondo Genet (37.2%) [21]. On the other hand, this finding is lower compared to the 89.9% reported in the same area 5 years ago [4]. The extreme reduction of the prevalence might be because of the implementation of a treatment programme targeting school age children in primary schools, provision of health education on how to prevent schistosomiasis, and community mobilization for latrine construction and utilization (*source-Sanja District health office*). Our finding is also lower than reports from Zarima (89.5%) [22], Wolaita zone (81.3%) [11], Methara (71.3%) [23], Tigray (56.5) [24] but higher than reports from Uganda (7%) [25], Jimma (24%) [13], WonjiShoa (8.8%) [26], and Chilga (15.5%) [27]. The reasons for prevalence variations could be due to the study periods, seasonal differences, treatments targeting deworming programs in schoolchildren, differences in the frequency of contacts with infested water, ecological distribution of snails, and climatic variabilities. In addition, differences in methods employed for stool examinations, sample sizes, and the variations in awareness regarding the transmission and prevention of* S. mansoni *infection may be the factors for the variations among findings.

This study revealed that males were more affected than females (42.1% versus 28%). Similar findings were reported in different parts of Ethiopia, such as Jimma [13] and Wondo Genet [12], and this may be due to increased exposure to infested water for bathing, swimming (≥3 times per week), washing clothes, and agricultural activities [4].

In the current study, PZQ at 40 mg/kg had a satisfactory effect on* S. mansoni*. The CR three weeks after treatment was found to be 90% (95% CI, 83.4%-96.5%). This is in line with those of studies conducted in South Côte d'Ivoire (88.6%) [8], in South Africa (88.07%) [28], and in other parts of Ethiopia (94%) [29]. On the other hand, the present finding is lower than the studies conducted in other parts of Ethiopia (99.1%) [13]. However, it is higher than the findings of studies conducted in Tanzania in which the cure rates among* S. mansoni* monoinfection,* S. mansoni* and HIV coinfected individuals were 62.6% and 48.3%, respectively [30]. The possible reason for the difference between of the current and the Tanzanian work could be differences in the duration of the posttreatment parasitological survey (12 weeks in Tanzania versus 3 weeks in the current study), making reinfection more likely in Tanzania. Moreover, intensity of infections, differences in sample sizes, skills of laboratory personnel, genetic diversity, and the intermittent nature of egg excretion by the parasites might have resulted in such differences.

The second therapeutic efficacy indicator of PZQ against* S. mansoni* infection was egg reduction rate (ERR), and 99.5% was reported in the present study. This was similar to the findings described from southwest Ethiopia (99.9%) [13] and South Côte d'Ivoire (98%) [31]. On the other hand, it is higher than that of a study done in Tanzania (77.2% in* S. mansoni* monoinfected and 75% in* S. mansoni*-HIV coinfected individuals) [30]. Similarly, a lower ERR (57.1%) was reported in Nigeria [32]. The possible explanation for the differences in ERR of the current study from that of Tanzania could be the aforementioned factors. However, the variation with the Nigerian study could be differences in the schistosoma species (it was against* S. haematobium*) in addition to the above factors.

After 6 months of posttreatment, a reinfection rate of 13.8% (95% CI, 5.9%- 21.9%) was observed. Similar reinfection findings were reported from South Africa (8.1%) [28] and Senegal (9.6%) [33]. Moreover, a study from Côte d'Ivoire showed a rapid and high reinfection rate a few weeks following treatment [34]. In this case, many factors might have resulted in the occurrence of high reinfection rates such as ecological and seasonal factors [8] and an area with high intensity of infection [35].

The prevalence and existence of reinfection rate of* S. mansoni* in the study area may suggest the burden of transmission and the current prevention and control challenges for the achievement of the planned goal of elimination of schistosomiasis-related morbidity in the country by 2020 [15].

The major limitation of this study is that infection intensity of* S. mansoni* was determined by examination of single stool specimen of each study participant. This might affect the accuracy of the egg detection and count of* S. mansoni*.

## 5. Conclusion

The finding indicated that schoolchildren in Sanja were at moderate risk for* Schistosoma mansoni* infection. The therapeutic potential of* Praziquantel* at 40 mg/kg against* Schistosoma mansoni* was efficient. However, the high reinfection rate in the six-month posttreatment period may indicate that the elimination and control of schistosomiasis might be challenged by several factors. Thus, the implementation of integrated snail control and preventive treatment strategies is important.

## Figures and Tables

**Table 1:** Sociodemographic and behavioral characteristics of study participants in Sanja town, Northwest Ethiopia, 2018.

Characteristics	Number	Percent
Sex		
Male	183	49.2
Female	189	50.8
Age in years		
8-11	157	42.2
12-14	215	57.8
Habit of washing or Bathing in river		
Yes	307	82.5
No	65	17.5
Open field defection habit		
Yes	261	70.2
No	111	29.8
Travel history to irrigation site		
Yes	183	49.2
No	189	50.8

**Table 2:** Prevalence of and intensity *S. mansoni* infection among schoolchildren in Sanja town, Northwest Ethiopia, 2018.

*Variables*	*Total Participants*	*Positive, n (*%)	*Infection intensity (epg), n (*%)		
			Light (<100)	Moderate (101-400)	Heavy (>400)
Sex					
Male	183	77 (42.1)	0 (0)	67 (87)	10 (13)
Female	189	53 (28)	0 (0)	45 (84.9)	8 (15.1)
Total	372	130 (35)	0 (0)	112 (86.2)	18 (13.8)
Age (years)					
8-11	157	72 (45.9)	0 (0)	61 (84.7)	11 (15.3)
12-14	215	58 (27)	0 (0)	51 (87.9)	7 (12.1)
Total	372	130 (35)	0 (0)	112 (86.2)	18 (13.8)

epg = eggs per gram.

**Table 3:** Cure rate of Praziquantel against *Schistosoma mansoni* infected schoolchildren in Sanja, North west Ethiopia, 2018.

Variables	Cure rate, n (%)		Total, n (%)	*χ* 2	P value
	Cured, n (%)	Uncured, n (%)			
Sex				11.293	0.186
Female	30 (88.2%)	4 (11.8%)	34 (100%)		
Male	42 (91.3%)	4 (8.7%)	46 (100%)		
Total	72 (90%)	8 (10%))	80 (100%)		
Age (years)				*χ* 2	P value
8-11	43 (93.48)	3 (6.52)	46 (100%)	28.019	0.923
12-14	29 (85.3)	5 (14.7)	34 (100%)		
Total	72 (90%)	8 (10%))	80 (100%)		

**Table 4:** Reinfection rate of S. mansoni infection six months after treatment in school children in Sanja, North west Ethiopia, May 2018 (n=72)

Variables	Re-infection status		*χ* 2	P value
	Reinfected, n (%)	Not reinfected, n (%)		
Sex			0.398	0.528
Female	3 (10.7%)	25 (89.3%)		
Male	7 (15.9%)	37 (84.1%)		
Total	10 (13.9%)	62 (86.1%)		
Age (year)			4.496	0.04
9	2 (18.2%)	9 (81.8%)		
10	2 (25.0%)	6 (75.0%)		
11	2 (20.0%)	8 (80.0%)		
12	3 (13.0%)	20 (87.0%)		
13	1 (11.1%)	8 (88.9%)		
14	0 (0.0%)	11 (100%)		
Total	10 (13.9%)	62 (86.1%)		

## Data Availability

All data generated or analyzed during this study are included in this published article.
